# An Up-to-Date Review of Biomaterials Application in Wound Management

**DOI:** 10.3390/polym14030421

**Published:** 2022-01-21

**Authors:** Adelina-Gabriela Niculescu, Alexandru Mihai Grumezescu

**Affiliations:** 1 Department of Science and Engineering of Oxide Materials and Nanomaterials, Faculty of Applied Chemistry and Materials Science, Politehnica University of Bucharest, 011061 Bucharest, Romania; adelina.niculescu@upb.ro; 2 Research Institute of the University of Bucharest—ICUB, University of Bucharest, 050657 Bucharest, Romania; 3 Academy of Romanian Scientists, 3 Ilfov Street, 050044 Bucharest, Romania

**Keywords:** biomaterials, wound management, wound dressings, semi-permeable films, foam dressings, hydroactive dressings, hydrocolloids, hydrogels, alginates, biomaterial scaffolds

## Abstract

Whether they are caused by trauma, illness, or surgery, wounds may occur throughout anyone’s life. Some injuries’ complexity and healing difficulty pose important challenges in the medical field, demanding novel approaches in wound management. A highly researched possibility is applying biomaterials in various forms, ranging from thin protective films, foams, and hydrogels to scaffolds and textiles enriched with drugs and nanoparticles. The synergy of biocompatibility and cell proliferative effects of these materials is reflected in a more rapid wound healing rate and improved structural and functional properties of the newly grown tissue. This paper aims to present the biomaterial dressings and scaffolds suitable for wound management application, reviewing the most recent studies in the field.

## 1. Introduction

**Figure 1:**
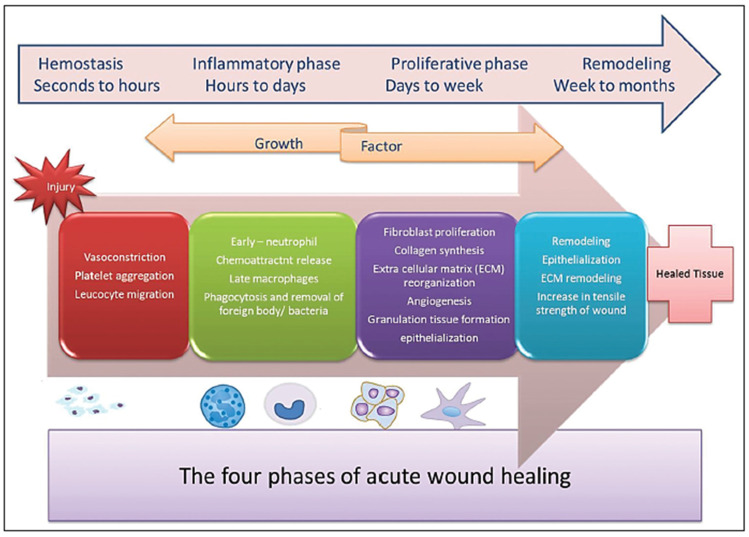
Distinct and overlapping phases of wound healing. Reprinted from an open-access source [20].

A wound represents the disintegration in the protective function of the skin, with or without loss of elemental connective tissue, as a result of thermal or physical damage [1,2]. Wound healing is a complicated multiphase and multifactorial physiological process that needs a suitable surrounding to achieve accelerated healing [3,4]. Once an injury occurs, the body starts a cascade of interrelated processes to repair the harmed tissues and restore the integrity of the skin [5,6]. Irrespective of what caused the damage, a similar multistep wound healing mechanism, counting hemostasis, inflammation, proliferation, and tissue remodeling stages, is put into action until complete wound closure [7,8,9,10,11] (Figure 1).

On the one hand, acute wounds can heal in a well-timed manner with a successful outcome (anatomic and physiologic restoration of the harmed area). On the other hand, in chronic wounds, the process fails time-wise, leading to incomplete restoration of skin structure and function [12,13,14]. The number of patients with chronic wounds increases every year [8]. As chronic wounds are only rarely present in otherwise healthy individuals, this phenomenon may be connected to the increase in life expectancy and prevalence of underlying conditions, such as diabetes and obesity [15,16,17]. As opposed to acute wounds, chronic wounds do not spontaneously heal, posing an aggravating potential that may escalate to amputations or even death [12,17,18]. Thus, it is of vital importance to ensure proper wound treatment, focused both on increasing the healing rate and improving the quality of healing [16].

Considering the different types of wounds and advancements in the biomedical field, a broad range of wound management options have appeared as solutions for efficient wound healing [19]. In this respect, this paper describes the wound dressings and biomaterial scaffolds developed in recent years, also taking into account the challenges, limitations, and future perspectives in the domain.

## 2. Types of Wounds

**Figure 2:**
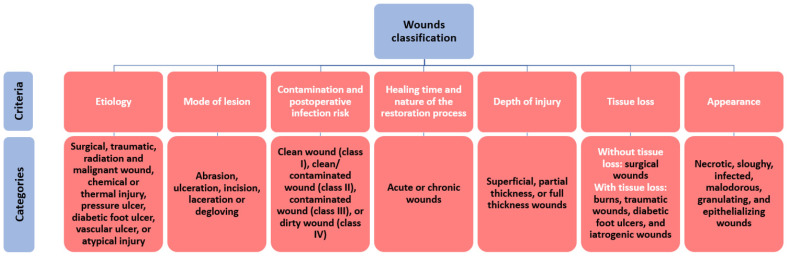
Wound classification. Created based on information from [21].

A wound can be defined as damage in the skin structure and function caused by external (e.g., physical, mechanical, thermal, chemical, electrical) factors or underlying medical or physiological disorders (e.g., diabetes, malignancies) [21]. Different attributes can characterize a wound, counting blood flow, oxygen, inflammation, edema, infection, wound metabolism, nutrition, innervation, repetitive trauma, previous injury handling, and systemic factors. Further, these characteristics help cumulate knowledge of the origin, pathophysiology, and condition of a wound [22], allowing its classification by several criteria (Figure 2), and permitting the assessment of appropriate treatment.

Among the various classification criteria, the most important one for choosing an adequate dressing and optimal wound management options is considering the healing duration and nature of the restoration process. An acute wound can heal completely, with minimum scar formation, without external support, in a healing time ranging from 8 to 12 weeks depending on the affected anatomical parts, size, and depth of the lesion. However, a chronic wound needs a longer time for healing, may reoccur, and display severe tissue scarring, necessitating specialized care [21]. Table 1 was created to emphasize the differences in the wound healing management of several distinct wounds.

## 3. Wound Dressings

Currently, a variety of dressings have been developed and applied in the clinic for treating all sorts of wounds. The benefits of using a wound dressing as a covering for damaged skin include maintaining a moist environment, absorbing excessive extracellular fluid, creating a barrier against infection, maintaining appropriate temperature, ameliorating pain, and cutting health care costs [3,8,24,25].

**Figure 3:**
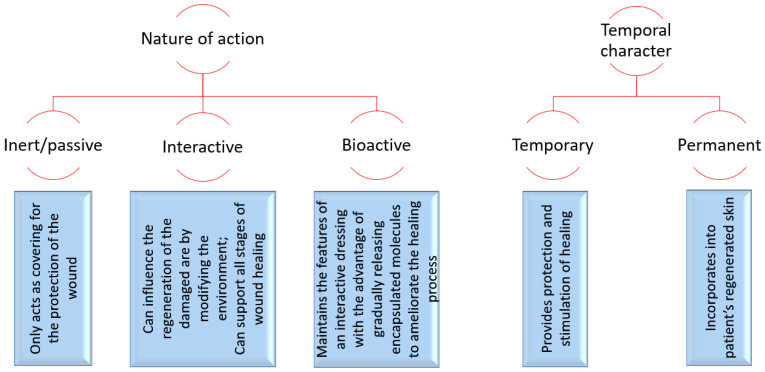
Wound dressing classification. Created based on information from [12,26,27].

Depending on their nature of action and temporal character, wound dressings can be classified and characterized, as shown in Figure 3.

**Figure 4:**
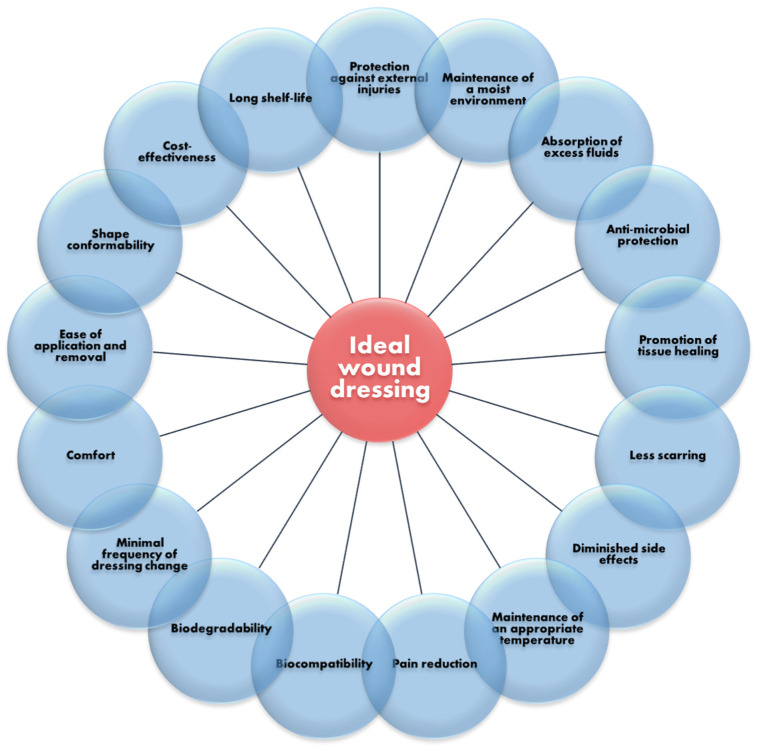
Main properties expected from an ideal modern wound dressing. Created based on information from [4,23,28].

Most of the traditional dressings fall under the category of inert dressings (e.g., gauze, cotton pads, bandages), being the least expensive and easiest to manufacture. Nonetheless, they have no antibacterial activity, face challenges in maintaining adequate moisture of the wound bed, and are prone to adhere to newly grown granulation tissue, thus causing pain upon removal. To overcome these drawbacks, research has been directed to developing multifunctional wound dressings that can simultaneously ensure the protection of the wounded area against external injuries and pathogens, be comfortable for the patient, reduce pain, increase healing rate, diminish scar formation, reduce care-associated costs, and have an extended shelf-life [3,4,23] (Figure 4).

Attempting to meet as many as possible of these characteristics, research has led to the production of modern wound dressings, such as semi-permeable films, foams, hydroactive dressings, hydrogels, hydrocolloids, alginates, and smart textiles [29,30]. In this respect, the following subsections aim to describe each of the mentioned categories of wound dressings and present their most recent advances.

### 3.1. Semi-Permeable Films

Semi-permeable film dressings are made of adhesive, thin, porous, transparent polyurethane that allows oxygen, carbon dioxide, and aqueous vapor transmission from the wound through the dressing. Concomitantly, these dressings are impermeable to bacteria, providing a barrier to external contamination. Film dressings are also endowed with autolytic debridement properties, suitable for epithelializing wounds and superficial wounds with low exudate amounts. Moreover, the transparency of polyurethane films is advantageous for wound inspection as it can be done without removing the dressing. On the other hand, the main drawbacks of these dressings are represented by their potential traumatic removal and the excessive pooling of exudate when used on heavily exuding wounds [23,31,32].

Several such wound management products have already entered the market, being currently used in the clinic in the treatment of various wounds (Table 2).

Nonetheless, ongoing research is focused on developing more performant film wound dressing alternatives, employing new biomaterial combinations, and endowing them with enhanced functionalities.

For instance, Rezaei Hosseinabadi et al. [37] have developed polyurethane dressings from two different polyols (i.e., castor oil and CAPA 7201). Both films presented smooth surfaces with no pores or cracks, but the CAPA-based polyurethane had higher crystallinity and lower thermal stability. There was no significant difference between the two formulations in terms of water vapor transmission rate. However, CAPA-based film exhibited higher water absorption capacity than castor oil polyurethane (5.67% vs. 0.76%), higher tensile strength (4 vs. 1.7 MPa), and higher elongation at break (550% vs. 100%). Moreover, its lack of toxicity, wound size reduction, and absence of in vivo adverse reactions recommend the CAPA-based polyurethane for use in wound management.

Despite polyurethane, other base materials can be used. For example, Ambrogi et al. [38] have developed alginate films containing pyrogenic silica-supported silver nanoparticles. These film dressings showed good hydration properties and a very slow silver release, leading to antimicrobial and antibiofilm activities against common wound-associated bacteria.

Alternatively, Rathore et al. [39] have fabricated biofilms with three different concentrations of chitosan (1%, 1.5%, and 2%) impregnated with ciprofloxacin to serve as dressings for infection-prone wounds. The dressings presented good texture and biocompatibility, also benefiting from a cost-effective and easy formulation. Moreover, the biomaterial films allowed a sustained drug delivery for wound healing, being effective against *Staphylococcus aureus*, *Escherichia coli*, and *Pseudomonas aeruginosa*, without affecting the NIH 3T3 fibroblast cell lines. In addition, the films exhibited suitable mechanical properties (tensile strength: ~40 MPa; elongation at break: up to 7% for 2% chitosan concentration; swelling: up to 290 ± 10% for 2% chitosan concentration) for wound dressing applications.

Different antimicrobial dressings were designed by Hubner et al. [40], who have prepared gelatin-based films containing different concentrations of glycerol and clinoptilolite zeolite impregnated with silver ions. The best results were obtained for 25% glycerol concentration and 0.5% zeolite concentration, leading to the slow release of the active compound and suitable physicochemical properties (tensile strength: 1.02 ± 0.05 MPa, elongation at break: 295 ± 19%, moisture content: 23.6 ± 0.2%) for the desired application. The researchers also obtained satisfactory bactericidal activity against *E. coli* and *S. aureus*, concluding that these films could serve as promising wound dressings that can ensure long-term antibacterial protection.

Another study by Akhavan-Kharazian et al. [41] proposed the improvement of mechanical properties of chitosan and gelatin film dressings by the incorporation of nanocrystalline cellulose (NCC) and calcium peroxide (CP) particles. The addition of these materials was also seen to reduce the water vapor transmission rate and swelling of the samples, while CP, in particular, was reported to increase the antibacterial activity of the dressings. Furthermore, the MTT assay results showed an increase in the growth of human fibroblast cells, demonstrating the biocompatibility and healing capacity of the newly developed dressings.

### 3.2. Foams

Foam dressings are solid porous matrices mainly made from polyurethane that can also be coated with a layer of soft silicone [32]. Foams can be sterilized and administered to wounds without causing any discomfort to the patient [42]. Polyurethane is particularly advantageous for wound dressings due to its softness, flexibility, optimal mechanical characteristics, air permeability, excellent absorption capability, and cost-effectiveness [43,44,45]. Moreover, the addition of a silicone membrane ensures exudate passage into the insulating foam, allows the dressing to remain in place, and protects the area from trauma when removing the dressing [32]. However, if factors, such as thickness, density, tensile strength, and elongation, of the freeze-dried foam are not adequately tuned, foams can produce discomfort and maceration of skin around the wound periphery. Another possible drawback of foams is the ingrowth of newly formed tissue into the dressing due to infrequent changes, leading to shearing trauma upon dressing removal [32,42].

Several foam dressings products that are already available on the market have been gathered in Table 3.

In addition to the commercially available products, recent research continues to design and investigate other foam-based wound dressings in the effort to create better wound management options.

In this respect, Namviriyachote et al. [43] have combined polyurethane foam dressings with different biomacromolecules, asiaticoside, and silver nanoparticles. At 2% incorporation, natural polymers (i.e., starches, high molecular weight chitosan, gelatin, and carboxymethyl cellulose) were able to improve the physicochemical and mechanical properties of the foam. Specifically, the first three biomacromolecules provided a stiffer and more porous structure, while carboxymethylcellulose exhibited the highest compression strength (15.29 ± 2.72 × 10^−3^ MPa) but reduced water vapor transmission capacity (0.76 ± 0.28 g/m^2^/min).

Alternatively, Pahlevanneshan et al. [53] have developed a porous nanocomposite polyurethane–nanolignin foam coated with ethanol extract of propolis (EEP). The addition of nanolignin and EEP increased the mechanical strength (tensile strength: 0.82 ± 0.09 MPa) and hydrophilicity (water contact angle: 50.1 ± 2.1°) of the dressing. Moreover, EEP endowed the foam with antibacterial properties. Overall, the dressing exhibited high cell viability and cell adhesion, promoting better skin wound healing than polyurethane alone.

Another example of an advanced polyurethane-based foam is offered by Bužarovska et al. [54]. The researchers employed soft thermoplastic polyurethane and zinc oxide to create highly porous nanocomposite foams. The foam dressing samples showed a low cytotoxic potential, good biocompatibility, adequate water vapor transmission (~8.0–8.9 mg/cm^2^/h), and the ability to support cell growth. Furthermore, the nanocomposite foams exhibited significant activity against Gram-positive and Gram-negative bacteria, being suitable candidates for active wound dressings.

Antibacterial foam dressings were also obtained by dos Santos et al. [55], who have deposited usnic acid-doped polyaniline on polyurethane foam. The scientists obtained strong antibacterial and antibiofilm (>90% reduction in biofilm adhesion degree) properties against *E. coli* and *S. aureus*, while the dressing also maintained its flexibility and intrinsic porosity. In addition to its efficiency, the material is also low-cost and eco-friendly, being a promising prototype for wound dressings.

Interesting results were also reported from the investigation of foams based on other biomaterials. For instance, Dutta et al. [56] propose using a topical hemostatic patch, called VELSEAL-T, at the bleeding wounded site of hemophilia patients. The proposed material is a chitosan-gelatin foam whose hydrophilic properties and porous structure facilitate large amounts of fluid absorption. Thus, blood is trapped inside the porous space of the foam where it comes in contact with thrombin and calcium chloride incorporated in the patch, facilitating clotting. By supplementary incorporation of tranexamic acid into the dressing, clot formation is reinforced, leading to rapid cessation of bleeding.

### 3.3. Hydroactive Dressings

Hydroactive dressings are multilayered polymer dressings that are based on a moist principle. They are similar to foams but absorb exudate in a different manner; they draw fluid into the polymer’s structure and trap the exudate to maintain a moist wound environment. This phenomenon is highly useful in preventing wound drying, promoting wound healing, and reducing the risk of maceration. Moreover, hydroactive dressings do not stick to the wound bed, thus lowering patient trauma on dressing removal, soothing painful wounds, and ensuring patient comfort and tolerability. In what concerns their limitations, hydroactive wounds are not recommended for lightly exuding and dry wounds [32,57].

Hydroactive dressings have shown promising results in the clinic, commercially available products being particularly effective in managing chronic wounds [57] (Table 4).

More recent research in the field of hydroactive dressings is not only focused on improving their properties but also on extending their applications to other types of wounds.

For instance, Yang et al. have investigated the use of a hydroactive dressing (i.e., DermaPlast Hydro #5353672) on the nasal ala of patients who underwent orthognathic surgery. Employing the hydroactive dressings for treating these wounds was seen to reduce the incidence of pressure injuries compared to adhesive tape, which was the standard of care in the control group. Nonetheless, the researchers suggested that additional studies are needed to confirm these results, recommending the conduction of a multisite randomized controlled trial to compare this dressing with other prevention methods.

One more example is offered by Ioffe et al. [63], who have evaluated the efficiency of a hydroactive dressing (i.e., HydroClean Plus) in the treatment of diabetic foot syndrome, particularly in patients with purulent-necrotic complications. The researchers reported an enhance in wound healing rate, achieving a complete cleaning of the affected area 6–7 days earlier than in the control group. The observation was confirmed by the results of cytological examination of the smears, leading to the conclusion that the used hydroactive dressing may be the optimal choice in managing this category of patients.

### 3.4. Hydrocolloids

Hydrocolloids are interactive occlusive, moisture-retentive dressings composed of two layers: a suspension of hydrophilic colloidal particles and a polyurethane layer that is impermeable to bacteria. Hydrocolloid dressings contain gel-forming agents (e.g., gelatin, sodium carboxymethylcellulose, and pectin) and other materials, such as elastomers and adhesive coatings. Thus, in the presence of wound exudate, this type of dressings forms a gel phase that enables moisturizing the wound and protecting the newly formed granulation tissue [31,32]. Other important advantages of hydrocolloids include pain-free removal, barrier properties against water, oxygen, or bacteria, and the promotion of angiogenesis and granulation [32].

Due to their beneficial properties for wound healing, several hydrocolloids entered the market and clinical facilities, showing good results in treating various wound types (Table 5).

Furthermore, research continued in the field, focusing on the development of more advanced hydrocolloid dressings, which involve new biomaterial combinations, provide enhanced functionalities, and allow synergistic treatment results when used together with other methods.

For instance, Wojcik et al. [67] have developed superabsorbent curdlan-based dressings acting as typical hydrocolloids. The authors used this natural polymer in combination with agarose and chitosan to create hybrid biomaterials suitable for the management of highly exuding wounds. The foam-like materials with a highly porous structure (66–77%) transformed into a soft gel in contact with the wounded area, exhibiting superabsorbent ability (1 g of biomaterial absorbs ~15 mL of exudate) and a proper water vapor transmission rate (1700–1800 g/m^2^/day) for optimal wound healing. The as-described dressings are non-toxic, stable in the presence of collagenases, biodegradable in lysozyme solution, and hinder fibroblast attachment. Taking into account the characteristics of the newly developed materials, the researcher concluded that they are promising dressing in the management of chronic wounds with moderate to high exudate.

A different wound management strategy was employed by Collado-Boira et al. [68], who have used sodium carboxymethylcellulose cellulose fibers (SCCFs) in combination with an extra thin hydrocolloid adhesive dressing in patients with peristomal skin lesions caused by severe irritant contact dermatitis. The as-described protocol showed promising results, reducing discomfort after 7 days and ensuring wound healing within 4 weeks.

Another synergistic approach is proposed for treating varicose ulcer wounds in patients with diabetes. According to Tănăsescu [69], by combined use of targeted antibiotic therapy, systemic treatment, local surgical treatment, and application of a hydrocolloid dressing, very good results can be obtained in a much shorter time than for conventional therapy. Moreover, colloidal-absorbent therapy is accessible through the polyclinic service or even at home.

### 3.5. Hydrogels

Hydrogels are complex three-dimensional structures composed of hydrophilic water-insoluble polymers that can absorb high water volumes (from 10% to thousands of times their equivalent weight). Thus, hydrogels present an excellent moisturizing ability and play a significant role in cleansing necrotic tissue. Moreover, hydrogels are generally transparent, offering the possibility for easy wound monitoring. Hence, hydrogel dressings can be employed in the treatment of various wounds, including burns, surgical wounds, pressure ulcers, and radiation dermatitis [23,32,70]. Unlike other modern wound dressings (e.g., foams, films, hydrocolloids), hydrogels also exhibit positive degradation properties, which permit the use of these materials as carriers when a targeted delivery of bioactive substances is required to the wound [31]. 

To emphasize their variety and versatility, a selection of commercially available hydrogels for wound management is presented in Table 6.

Regarding their disadvantages, the main drawback of plain hydrogels is the poor bacterial barrier properties [32]. Thus, recent research was directed towards improving their antimicrobial activity by incorporating different nanomaterials into the dressings [44].

For instance, silver nanoparticles (Ag NPs) were the antimicrobial agent of choice for Nešović et al. [77], who have embedded them into chitosan and polyvinyl alcohol hydrogels. The resulting nanocomposite dressings presented excellent physicochemical properties, appropriate swelling and silver release profiles, and strong antibacterial activity against *E. coli* and *S. aureus*. Similarly, Diniz et al. [78] have incorporated Ag NPs into sodium alginate/gelatin hydrogels for healing cutaneous lesions. The authors reported promising in vivo results, counting the ability of the dressing to reduce wound size, promote earlier development and maturation of granulation tissues, and significant bactericidal activity against *P. aeruginosa* and *S. aureus*. Ag NPs were also used by Gupta et al. [79]. The researchers prepared Ag NPs using curcumin-cyclodextrins loaded into bacterial cellulose-based hydrogels, obtaining high cytocompatibility, moist wound-healing properties, and potent antimicrobial activity against common wound-infecting pathogens.

Another strategy approached in recent studies consists of zinc oxide nanoparticle (ZnO NPs) addition. For example, Khorasani et al. [80] have loaded ZnO NPs into heparinized polyvinyl alcohol/chitosan hydrogels. ZnO NPs addition was noted to improve the mechanical and thermal properties of the hydrogel dressing, ensure the sustained release of heparin, and endow the material with antibacterial properties. Alternatively, Raafat et al. [81] have embedded ZnO NPs into a xanthan/polyvinyl alcohol-based wound dressing hydrogel. The as-prepared material showed a homogenous porous structure that, which, along with the presence of ZnO NPs, contributes to the control of fluid uptake ability, water retention, and water vapor transmission rate. Moreover, the nanocomposite hydrogel displayed an efficient microbial barrier potency, with strong activity against *S. aureus*, *E. coli*, and *C. albicans.*

An innovative hydrogel dressing was proposed by Kudinov et al. [82], who fabricated an easy-to-use placental mesenchymal stromal cell (MMSC) secretome-based chitosan hydrogel for the treatment of *S. aureus*-infected burn wounds. After testing this novel dressing, the researchers reported almost complete epithelialization, with high levels of vascularization and angiogenesis. MMSC secretome was found to have a similar antimicrobial activity to that of Miramistin and Bepanthen Plus, and the ability to secrete factors that can promote skin healing in all regeneration phases. Thus, the authors recommend the translation of this hydrogel into clinical practice as its preparation is rapid and simple. However, the obtained experimental data requires further research and clarification before moving to human use.

### 3.6. Alginates

Alginates (i.e., calcium or calcium sodium salts of alginic acid) are naturally occurring polymers that have been separated in the literature into a distinct category of dressings [83]. When applied to a wound, they form a hydrophilic sodium alginate gel as the calcium present in the material’s structure reacts with the sodium salts present in the wounded area. The formed gel further absorbs the fluid at the injury site, providing a moist environment for the wound to heal optimally [23,32,44,84,85,86]. Other advantages of alginate dressings include their easy removal, hemostatic properties, flexibility, permeability to water vapor, carbon dioxide, and oxygen, and protection against bacterial infections. On the other hand, several drawbacks must be taken into consideration when using alginates for wound management. Specifically, alginates are non-adhesive, and they may provoke allergic reactions in individuals allergic to seaweed-derived products or when the exudate amount is not enough for forming the removable gel [32,42,87].

Nonetheless, these disadvantages can be overcome or avoided in practice by matching the dressing to suitable types of wounds and adding a secondary dressing to hold it in place. Thus, several alginate products have already entered the market and have been used in the clinic with good results (Table 7).

In addition to the commercially available products, ongoing research focuses on designing enhanced alginate dressing formulations, considering the advantageous properties that emerge at the convergence of this natural polymer with other biomaterials, nanoparticles, and/or biomolecules.

As an example, Zhao et al. [90] have coated a calcium alginate dressing with a mixture of high and low-molecular-weight chitosan. After testing the alginate-based samples, the researchers noted good moisturizing and antibacterial properties, inhibition of inflammation, promotion of wound healing, and non-cytotoxicity.

An interesting strategy for treating diabetic foot ulcers (DFU) via alginate-based dressings is proposed by Wang et al. [91]. The scientists created an alginate wound dressing containing magnesium and hydroxypropyltrimethyl ammonium chloride chitosan, which presented good prospects for clinical translation. Specifically, the dressing showed good biocompatibility, accelerated DFU healing, and effectively eradicated antibiotic-resistant bacteria, such as methicillin-resistant *S. aureus* and methicillin-resistant *S. epidermidis*.

A different approach was taken by Liang et al. [92], who have fabricated an oxidized sodium alginate sponge functionalized with polydopamine/silver composite nanospheres. The functionalization contributed to improving the stability of the sponge without compromising its porosity. Moreover, the as-described dressing exhibited high blood compatibility, low cell cytotoxicity, good hemostatic performance, and strong antimicrobial activity against *P. aeruginosa*, *S. aureus*, and *E. coli.*

Alternatively, Azam et al. [93] have developed a novel alginate-based material by adding 2-deoxy-D-ribose to clinically used alginates. The dressings showed more than 90% release of the sugar in the first three days, followed by a lesser and sustained release for up to eight days. Moreover, sugar addition was observed to significantly increase wound healing rate when compared to both the control group and the group treated with pristine alginate.

### 3.7. Smart Textiles

For decades, people have been using textile wound dressings as protectors against pathogens and external injuries. Nonetheless, improving these materials and creating advanced wound management options is still of great interest nowadays [94,95,96,97].

In the context of designing smart textiles, electrospun nanofibers have drawn tremendous scientific attention, especially due to the possibility of loading bioactive molecules within the nanofiber [98,99,100]. Thus, optimal burst control and enhanced drug stability can be achieved, contributing to the proper healing of the wounded area. Moreover, nanofibers can mimic the ECM, furnishing a highly porous structural support for growing cells and subsequentially accelerating skin healing [101,102]. For instance, Amanzadi et al. [103] have fabricated a multilayer electrospun chitosan-based dressing containing *Semellil Melilotus Officinalis* extract. The dressing consists of three layers: a protective polyurethane nanofibers layer, a layer of chitosan nanofibers loaded with extract, and a chitosan mat to improve the sustained release. In vivo tests on rat models revealed that the as-described textile dressing leads to an improved wound healing profile, with a wound-closure percentage of 94% after 14 days. A similar multilayer construct is proposed by Shokrollahi et al. [104]. The researchers have combined a chamomile-loaded carboxyethyl chitosan and polyvinyl alcohol nanofibrous layer (in contact with the wounded area) and a poly(ε-caprolactone) nanofibrous layer (strength enhancer) to create a hybrid mat for wound care. The newly developed material showed satisfactory tensile strength (8.2–16.03 MPa), antioxidant properties (6.60–38.01%), high antibacterial efficiency, proper cell viability, and sustained chamomile release. Alternatively, Ahmadian et al. [105] have created a novel antibacterial electrospun nanofiber mat composed of ethyl cellulose, poly lactic acid, and collagen nanofibers incorporated with silver sulfadiazine. The dressing showed inhibitory properties against *Bacillus* (9.71 ± 1.15 nm) and *E. coli* (12.46 ± 1.31 nm) bacteria while improving cell proliferation and adhesion without imparting any cytotoxic effect on NIH 3T3 fibroblast cells. Thus, these mats are considered suitable for application as dressings in wounds necessitating infection control.

Another approach to designing advanced textile dressings involves the addition of micro and nanoparticles. As an example, Melamed et al. [106] have investigated copper oxide microparticle (COD)-impregnated wound dressings for the treatment of wounds in diabetic patients. The authors reported that COD significantly influenced the healing of hard-to-deal diabetic wounds, providing antibacterial effects, and directly enhancing the process. Similarly, Deokar et al. [107] have used copper oxide and zinc oxide to coat bandages, endowing them with antimicrobial properties. The authors used both water and ethanol-based syntheses, concluding that the ethanol-free bandages are safer to use (resulting in a lesser impact on embryo development) and safer to manufacture (reducing ignition risk in bulk scale production). As demonstrated by Majumder et al. [108], ZnO nanoparticles can bring synergistic properties to hybrid dressings as well. The researchers developed a biomimetic composite by grafting a hydrogel on silk fibroin fabric and functionalizing it with metal oxide NPs. The resultant wound dressing had sufficient water-vapor permeability (480 g/m^2^/day), adequate mechanical properties (tensile strength: 22.5 ± 1.3 MPa; extension at break: 6 ± 0.69 mm), and strong antibacterial activity, being a promising material for wound management and regenerative medicine.

A different strategy is proposed by Akolpoğlu Başaran et al. [109], who have encapsulated heparin into poly(lactic-co-glycolic acid) (PLGA) nanoparticles, which were further incorporated into sericin/gelatin nanofibers during electrospinning. The researchers tested several protein ratios in the search for the optimum formulation able to ensure controlled cargo release and help skin tissue regeneration. The study obtained the most promising results for the dressing with a sericin/gelatin ratio of 1/2, which presented proper fiber morphology, a high water retention degree (~7.5 after 7 days at physiological pH), and a low degradation degree (6.95 ± 4.52 % cumulative weight loss after 7 days), concluding that these constructs can be safely used for skin tissue engineering.

### 3.8. Other Dressings

Recent studies have also reported interesting results for dressings that do not fall under any category presented above. For instance, Choi and Jeon [110] have fabricated functional superabsorbent sponges made of natural polymers. The authors prepared an alginate/carboxymethyl cellulose-embedded dextran hybrid dual layer constructs via the freeze-drying method. The obtained materials could absorb large amounts of moisture (up to 1800% swelling ratio), provide morphological stability through proper tensile strength (up to 45 kgf/cm^2^), and uniform porosity. Encouraged by these results, the authors stated that combining this newly developed sponge with antibacterial agents could generate antimicrobial bandages for wounds necessitating the absorption of high levels of blood and body fluids.

A different sponge-type dressing was developed by Choi et al. [111], who embedded sustained oxygen-releasing PLGA microspheres into an alginate-based hydrogel. The as-described oxygen-releasing hydrogel sponge induced neovascularization and promoted cell proliferation, thus facilitating effective wound healing. In particular, this dressing is suitable for supplying oxygen to deprived cells and tissues to enhance angiogenesis in the wounded area.

Alternatively, Kaur et al. [112] have fabricated polyvinyl alcohol (PVA)-sodium alginate membrane dressings for the topical delivery of bacteriophages (i.e., MR10 phage) and antibiotic (i.e., minocycline) to infected burn wounds. In vitro studies revealed self-adherence, antibacterial properties, and biocompatibility of the developed membrane, while in vivo tests on an MRSA-infected murine burn wound model displayed significant pathogen reduction, wound contraction, and diminished inflammation.

Antimicrobial dressings have also been prepared by incorporating beehive products as antibacterial and wound-healing agents. As an example, Tang et al. [113] have incorporated honey into an alginate/PVA-based nanofibrous membrane. The membrane dressings exhibited non-cytotoxicity, biocompatibility, and enhanced antioxidant activity, being able to control reactive oxygen species overproduction. Moreover, honey-loaded nanofibers reportedly inhibited bacterial growth, their antimicrobial activity increasing with the increase of honey content. The dressings with the highest content of honey (20%) led to an inhibition zone diameter of 11.38 ± 0.42 mm for *E. coli* and 13.67 ± 1.29 mm for *S. aureus*.

Differently, Eskandarinia et al. [114] have proposed a bilayer wound dressing comprising a dense polyurethane/ethanolic extract of propolis (PUR/EEP) membrane and a polycaprolactone/gelatin (PCL/Gel) nanofibrous scaffold. The PUR/EEP layer protects the wounded area from external contamination and dehydration, whereas the PCL/Gel represents the sub-layer responsible for cell adhesion and proliferation. Overall, the bilayer structure offers potent antimicrobial activity, with diameters of inhibition zones of 5.4  ±  0.3 mm against *S. aureus*, 1.9  ±  0.4 mm against *E. coli*, and 1.9  ±  0.4 mm against *S. epidermidis*. Furthermore, the dressing showed high hydrophilicity (51.1 ± 4.9°), biodegradability, and biocompatibility; in vivo tests revealed a significantly improved wound closure rate and collagen deposition. Additionally, the mechanical properties (tensile strength: 5.6  ±  0.6 MPa; elongation at break: 333.2  ±  12.4%) also recommend these constructs for wound management applications.

## 4. Biomaterial Scaffolds

If not treated promptly and adequately, wounds, burns, and injuries can get infected and extend on a large surface or into deeper tissues. In such cases, a wound dressing might not be sufficient for ensuring proper healing [115]. Thus, spatial reconstruction by means of biomaterial scaffolds is often implied [15]. Ideally, such constructs should be able to sustain both aesthetic and complete function tissues, imparting shape, mechanical support, and proper microarchitecture for cellular growth and reorganization to improve and stimulate the healing process [116,117].

**Figure 5:**
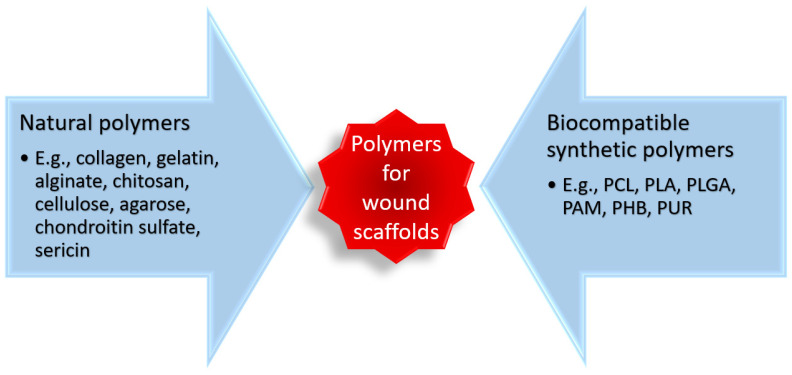
Classification and examples of polymers used for fabricating biomaterial scaffolds. Created based on information from [115,118,120,121,122,123,124,125,126]. Abbreviations: PCL—poly (ε-caprolactone), PLA—polylactic acid, PLGA—poly(lactic-co-glycolic acid), PAM—polyacrylamide, PHB—poly-3-hydroxybutyrate, PUR—polyurethane.

To create performant biomaterial scaffolds, much attention has been drawn to polymer-based materials (Figure 5) that can be fabricated into diverse structures, with various configurations, degradation rates, and different drug delivery kinetics [118,119,120,121].

To date, collagen is the most used biopolymer for wound healing, as numerous scaffolds available on the market are based on this material [27,118,127,128]. Nonetheless, recent research started focusing on various other biomaterials too.

For instance, bacterial cellulose (BC) has attracted scientific interest in developing innovative scaffolds. Cherng et al. [125] have evaluated the potential of BC-based scaffolds of epithelial regeneration and wound healing in vitro and in vivo. The authors reported excellent biocompatibility in vitro, as the material was able to maintain the stemness function of cells and promote keratinocyte differentiation. Moreover, the promising results were also noted in vivo, including improved ECM deposition and controlled excessive inflammation, concluding that the tested scaffolds are suitable candidates for skin injury repair.

Chitosan is another highly researched material, especially for scaffolds able to perform drug delivery beyond their physical support role [19]. In this respect, Castillo-Henriquez et al. [124] have fabricated a chitosan-based thermo-responsive scaffold that can sustain the release of Dexketoprofen trometamol for 24 h. The developed scaffold can also reduce side effects of the drug, overcoming adherence issues and potential wound healing complications. Choudhary et al. [129] have also used this natural polysaccharide, creating chitosan-reinforced graphene-silver-polycationic peptide nanocomposites for wound healing application. The scaffolds displayed efficient antibacterial properties, unique mechanical properties, and excellent porosity, fluid absorption, and blood clotting capacity, representing viable solutions for trauma care management.

According to Napavichayanun et al. [126], interesting results were also obtained for biomaterial scaffolds made of agarose and sericin additivated with plasticizers. By the combined use of freeze-thawing and freeze-drying methods, there was obtained strong bonding between sericin and other components, resulting in a low swelling ratio and low protein release of the scaffolds, which are advantageous properties for developing controlled drug release scaffolds. In addition, the scaffolds were able to activate cell migration towards accelerating the healing process, while the plasticizers contributed to the enhancement of scaffold elasticity.

A different example is offered by Laiva et al. [123], who have developed a collagen-chondroitin sulfate scaffold functionalized with nanoparticles carrying an anti-aging gene, β-Klotho, on human adipose-derived stem cells (ADSCs). By studying this biomaterial, it was established that it ensures controlled activation of ADSC’s regenerative abilities, enhances activation of transcription factor Oct-4, increases the expression of the anti-fibrotic gene TGF-β3, controls human endothelial angiogenesis and pro-fibrotic response in dermal fibroblasts, enhances regeneration of the basement membrane, and decreases the levels of scar-associated α-SMA protein with improved qualitative elastin matrix deposition. Overall, the identified properties highly recommend this scaffold for wound healing applications.

However, promising results were also obtained for cell-free scaffolds, as is the case in the study conducted by Gerges et al. [122]. The authors have designed a biodegradable polyurethane-based scaffold for soft tissue regeneration. The working principle was noted to be the gradual infiltration of undifferentiated mesenchymal stem cells from the periphery to the center of the scaffold, followed by the rapid formation of a functional vascular network supporting cell viability over time. Moreover, the scaffold was reported to preserve balanced physicochemical properties, with an exceptional combination between softness and resilience. Aside from polyurethane, poly (ε-caprolactone) (PCL) is another promising synthetic polymer, highly researched for wound healing applications due to its availability, cost-effectiveness, biological properties, and mechanical strength [130]. Considering these advantageous features, Ahmed et al.(2021) [131] have used PCL to fabricate nanofibrous scaffolds incorporated with magnetite nanoparticles doped with different concentrations of silver ions. By making the magnetite structure more defective, silver modified the interface with the polymer, promoting the protrusion of the nanoparticles from the surface of the PCL nanofibers. Thus, the roughness and hydrophilicity of the material increased, positively impacting cell adhesion and growth. It was noted that the viability of human melanocytes, antibacterial activity against *E. coli* and *S. aureus*, and skin wound healing rate increased with the increase of silver in the magnetite phase of the scaffolds. The in vivo healing rate was enhanced to over 50% for the scaffold without silver ions and to over 90% for the scaffolds with the highest concentration of silver. Moreover, the samples containing the highest concentration of silver had a tensile strength of 4.42 ± 0.25 MPa, a strain at break of 147.4 ± 3.4%, a toughness of 4.25 ± 0.33 MJ/m^3^, and a porosity of 87.93 ± 3.1%. Therefore, the mechanical properties of the obtained composite material were fit for use in wound management and reconstructive skin therapies.

PCL was also reported as promising in combination with other polymers. For instance, Ahmed et al. (2020) [132] have created a nanofibrous blend matrix of cellulose acetate and PCL into which they incorporated various metallic nanoparticles (i.e., ZnO, Ag, and CuO). The scaffolds were proven suitable candidates for wound disinfection and dressing applications, exhibiting antibacterial activity against *S. aureus* and *E. coli*. The highest inhibition zone was obtained in the case of Ag-loaded scaffolds, with diameters of around 12.3 ± 2.2 nm for *S. aureus* and 11.2 ± 1.5 nm for *E. coli*; however, CuO incorporation led to close results: 9.4 ± 1.2 nm and 10.1 ± 1.5 nm, respectively. Alternatively, Sadeghianmaryan et al. [133] have fabricated scaffolds with homogenous and soft polyurethane nanofibers containing different amounts of PCL and nanographene oxide. All the spun scaffolds exhibited a high level of porosity (~90%), with the fiber diameter increasing as the graphene oxide concentration increased. The addition of graphene oxide to the polymer scaffolds resulted in good biocompatibility to skin fibroblast cells and increased hydrophilicity, demonstrating their potential use in skin tissue engineering.

A distinct scaffold is proposed by Guha Ray et al. [134]. The researchers modified eggshell membranes (ESM) using chitosan/PCL nanofibers towards manufacturing a bilayered scaffold. The scaffold presented a biomimetic architecture and composition, which facilitated extensive cell adhesion, migration, and proliferation. The presence of ESM led to a natural adhesion of the scaffold to the wound bed while implanted on an in vivo full-thickness wound. Compared to bare ESM, the polymer-modified scaffolds conducted to faster re-epithelialization and dermal regeneration with collagen deposition. Thus, it was concluded that the bilayered composite is a potential dermal substitute.

Polylactic acid (PLA) has also been utilized in various combinations to obtain innovative biomaterial scaffolds. As an example, Hajikhani et al. [135] have fabricated PVP/PLA-PEO complex nanofibers loaded with collagen and cefazolin. The authors reported that collagen doses of 10% and 20% led to significantly increased healing speed, while the samples containing 40% collagen produced a decrease in wound healing rate in mice. Moreover, the scaffolds presented antimicrobial properties, effectively inhibiting microorganisms’ growth. Similarly, Fatahian et al. [136] have developed PVA/PLA nanofibrous scaffolds encapsulated with ceftriaxone (antimicrobial agent) and tranexamic acid (coagulant). The scaffolds showed more than 90% efficiency against *E. coli* and *S. aureus*, acceptable blood coagulation ability with an average absorption of ~0.04 nm, gel formation ability of about 45 min, and successful cell proliferation.

A different approach is offered by Yu et al. [137], who have manufactured poly(lactic-co-glycolic acid)/gelatin (PLGA/Gel) nanofibrous scaffolds incorporated with liraglutide (Lira) for skin tissue engineering. The scientists observed that the addition of Lira to the scaffold increased its pore size, hydrophilicity, elasticity, and degradation properties. The nanofibrous composite was evaluated on diabetic dermal wounds, showing considerably improved healing efficiency described by shortened wound closure time, increased blood vessel density, and enhanced collagen deposition and alignment. Thus, it was concluded that PLGA/Gel loaded with Lira represents a promising strategy to accelerate diabetic wound repair.

Another interesting scaffold for diabetic wounds was created by Sanhueza et al. [138], who have prepared poly-3-hydroxybutyrate (PHB) and gelatin fibrous constructs. In vitro, the scaffolds exhibited excellent fibroblasts viability and attachment after incubation for 1, 3, and 7 days. In vivo tests followed wound healing in diabetic rats for 21 days, leading to the observation of faster healing for gel-containing scaffolds, while the PHB-Gel treated wounds were reported to be in a late proliferative stage, with higher content of hair follicles and sweat glands and lower content in fibroblasts compared to the control wounds.

Recently, pH-sensitive polymeric scaffolds started to gain interest in wound healing; their efficacy resides in the fact that the normal human skin pH is between 4 and 6, while, in the case of an injury, it raises the physiological value [139]. In this context, Garg et al. [140] have developed a pH-sensitive scaffold made of polyacrylamide (PAM)-grafted flax seed mucilage graft copolymeric hydrogel. The scaffold showed maximum swelling at 7.4 pH, tissue compatibility, satisfactory fibroblast growth, and sufficient collagen deposition, being considered promising materials for wound management.

## 5. Challenges and Limitations

Modern wound management has come a long way from traditional wound dressings, such as bandages and gauzes, which require a regular application, have poor adhesion properties, cannot ensure proper wound drainage, and may even cause pain upon removal [31]. Nonetheless, there is still no ideal wound dressing, neither on the market nor in research facilities, that can universally fit and heal all wound types.

The effectiveness of first-line interactive/bioactive dressings is poorly assessed as evidence is still limited to only a few clinical studies. Thus, there are needed more high-quality randomized controlled trials that can bring certain confirmation on the performance of dressing products to better comprehend the state-of-the-art in the field [32].

Moreover, challenges arise in choosing the best-fitted dressing for a specific wound. Even with modern wound management options, special consideration must be given to the patient’s primary disease and the physiological mechanisms of the wounds. Other dressing selection challenges may be related to legal, technical, methodological, and financial concerns that limit the possibility of conducting high-quality evidence-generating studies, hindering the translation of newly developed dressing into clinical practice [23,32].

In addition, clinicians and managers should be able to make more informed decisions on the costs of materials and procedures and the effects on the financial budgets of both patients and healthcare facilities. Therefore, wound management strategies should be developed in the direction of minimizing the costs while maintaining optimal clinical outcomes [141]. Better collaboration should be encouraged between the industry, clinical research, and clinical practice market segments to attain such perspectives.

## 6. Conclusions and Future Perspectives

To summarize, wounds of all sorts can occur throughout anyone’s life, requiring prompt and efficient care to avoid potential complications. Thus, wound management continues to be a topic of high interest in the medical field, aiming to develop better biomaterial formulations for various dressings and scaffolds. A broad range of semi-permeable films, foams, hydrogels, hydrocolloids, hydroactive dressings, alginates, and biomaterial scaffolds have been reported in the literature with different degrees of success in wound healing, while several such products have already managed to enter the market and are currently used in the clinical practice.

Nonetheless, the search for optimal wound management options concerns ongoing interdisciplinary research studies. Some of the most emerging perspectives in the field use localized nucleic acids delivery for the treatment of nonhealing chronic wounds [142], application of cold atmospheric pressure plasma treatment alongside temporary skin replacement scaffolds [143], and dosing topical formulations for wound dressings via 3D printing [144]. Moreover, the advancements encountered in the usage and regulation of stem cells paves the way for designing smart healing tools that lead to natural mimicking outcomes, where factors, such as pigmentation, epidermal appendages, vascular plexus, and subcutaneous tissues, are also restored [145].

To conclude, despite the intense research in the field, there is still room for improvement towards creating performant multifunctional dressings and scaffolds. Wound management optimization should be achieved starting from the design, materials choice, and dressing selection for specific wound types. Only through a clear understanding of the current state-of-the-art, challenges, limitations, and development perspectives, better solutions can be envisaged for effective personalized wound care.

## Figures and Tables

**Table 1:** Correlation between various wounds and their appropriate dressing. Adapted from an open-access source [23].

Wound Type	Characterization	Examples of Appropriate Types of Dressings
Diabetic foot ulcer	Caused by neuropathy and lower extremity vascular disease	Silver ion foam dressings, hydrofiber dressings, non-adhesive dressings
	Lacks oxygen and blood supply in the wound bed	
	Long-term stagnation in the inflammatory phase	
Chronic venous leg ulcer	Caused by the high pressure of the blood in the leg veins	Alginate dressings, silver-impregnated dressings, foam dressings
	Lacks blood supply in the wound bed	
	Large amount of necrotic tissue	
	Abnormal exudate on the ulcer surface	
	Accompanied by multiple bacterial infections	
Pressure injury	Caused by stress and tissue tolerance	Foam dressings, hydrocolloid dressings, polyurethane film
	Local injury to the skin or subcutaneous soft tissue	
	Occurs at the site of the bone prominence or due to the compression of a medical device	
Radiation dermatitis	Local skin lesions caused by radiation	Film dressings, silver-containing hydrofiber, polyurethane foam, alginate dressings
	Slow cell proliferation	
	Decreased cytokine activity	
	Decreased collagen content	
Burn and scald	Tissue damage caused by heat	Moist occlusive dressings, silver-impregnated dressings
	Large amount of exudate	
	Prone to infection	
	Severe cases can affect subcutaneous and submucosal tissues	

**Table 2:** Examples of commercially available film dressings.

Commercial Product	Dressing Type	Observations	Refs.
Hydrofilm	Thin, sterile, semi-permeable film dressing	Transparent dressing	[32,33]
		Waterproof	
		Bacteria-proof	
		Sticks to the skin by a hypoallergenic acrylic adhesive	
		Suitable as primary dressing for post-operative and trauma wounds or as a secondary dressing for retention purposes	
		Can remain on the skin for several weeks without causing trauma upon removal	
Hyalosafe	Hyaluronic acid-based film	Transparent dressing	[34]
		Allows easy wound monitoring	
		Suitable for treating moderate exuding wounds and surgery wounds	
OpSite	Thin semi-permeable film dressing	Covered with hypoallergenic acrylic derivatives	[32,35]
		More porous and permeable to water vapor and gases, but not to exudate	
		Suitable for relatively shallow wounds	
Tegaderm	Semi-permeable adhesive sterile film	Permeable to water vapor and oxygen	[36]
		Provides a moist environment that enhances healing rate	
		May be useful in preventing skin breakdown at pressure areas	
		Suitable for different wounds, including donor sites, minor burns, abrasions, and lacerations	

**Table 3:** Examples of commercially available foam dressings.

Commercial Product	Dressing Type	Observations	Refs.
Allevyn	Hydrocellular foam dressing	Able to absorb, retain, and transpire to achieve optimal fluid balance	[32,46,47]
		Promotes faster healing by maintaining an optimal environment	
		Reduces maceration risk by not allowing the wounded area to become too wet	
		Common choice for managing donor site wounds	
Betafoam	Povidone-iodine foam dressing	Effective antimicrobial activity with minimal cytotoxicity to host cells	[46,48]
		Better ease of use, less bleeding, and adherence on removal of dressing, less leakage of exudate compared to commonly used dressings	
		Superior fluid-handling capacity (e.g., improved moisture retention, fast fluid absorption time)	
		Better wound-healing efficacy than conventional mesh dressings	
Cavi-care	Foam dressing	The porous structure allows the maintenance of a moist environment to promote healing, while permitting excessive exudate to be drained through the dressing	[32,49]
		Helps avoid secondary infection and offensive smell	
		Suitable for cavity wounds, auricular pressure wounds, and following primary non-glandular hypospadias repair or syndactyly correction	
Mepilex	Silver-impregnated foam dressing	Excellent antimicrobial activity against common wound pathogens	[32,50,51,52]
		Absorbs exudate and maintains a moist wound environment	
		Self-adherence properties can cut treatment costs by reducing the need for frequent dressing change	
		Suitable for a variety of wounds, including surgical wounds, pressure injuries, and burns	
Permafoam	Foam dressing	Has a gas permeable, waterproof, and germ-resistant outer layer	[32,36]
		Absorbs exudate to create a moist environment	
		Painless on removal	
		Leaves no residue	
		Suitable for moderate to high exuding wounds	
		May be used as a secondary dressing	

**Table 4:** Examples of commercially available hydroactive dressings.

Commercial Product	Dressing Type	Observations	Refs.
Biatain Ag	Hydroactive silver-impregnated foam-like dressing	Soft, absorbent dressing	[32,58,59,60]
		Exerts antimicrobial activity due to incorporated silver	
		Superior performance than its non-silver counterpart in terms of relative ulcer area reduction and healing rate	
		Can be used in the treatment of hard-to-heal venous leg ulcers	
Cutinova Hydro	Hydroactive foam-like dressing	Self-adhesive polyurethane gel matrix embedded with highly absorptive granules	[32,61]
		Allows a fluid uptake of 10 times its weight	
		Allows the loss of water vapor from the dressing and transmission of oxygen through it to the wounded area	
		Effective in debriding slough and necrotic tissue	
		Can be used in the treatment of leg ulcers, pressure ulcers, traumatic wounds, and diabetic foot ulcers	
Tielle	Hydroactive foam-like dressing	Has additional wound contact layers to avoid adherence when the wound is dry	[32,35,62]
		The occlusive polymeric backing layer prevents excess fluid loss and bacterial contamination	
		Can be used on venous leg ulcers, pressure ulcers, and other similar wounds	
		Can act both as a primary and secondary dressing	

**Table 5:** Examples of commercially available hydrocolloid dressings.

Commercial Product	Dressing Type	Observations	Refs.
Comfeel	Hydrocolloid dressing	Promotes healing and reduces patients’ discomfort	[64,65]
		Absorbs at least two times its own weight	
		Can be used for various wounds, including severe friction burns, gravel rash, and following excision of pilonidal sinuses	
DuoDerm	Hydrocolloid dressing	Occlusive dressing, impermeable to water, water vapor, oxygen, and bacteria	[36]
		Provides pain relief by keeping the nerve endings moist	
		The moist environment promotes debridement of sloughy tissue and facilitates granulation	
		Patients can bathe with the dressing in situ	
		Helps prevent skin breakdown at pressure areas	
		Suitable for superficial wounds with light to moderate exuding	
Granuflex	Hydrocolloid dressing	Occlusive dressing, impermeable to water, water vapor, oxygen, and bacteria	[36]
		Provides pain relief by keeping the nerve endings moist	
		The moist environment promotes debridement of sloughy tissue and facilitates granulation	
		Patients can bathe with the dressing in situ	
		Suitable for light to moderate exuding wounds	
		Can be applied to partial or full thickness wounds	
OxyBand	Self-contained multiple layers hydrocolloid dressing	Oxygen prefilled wound dressing	[58,66]
		The top layer has a waterproof barrier film	
		Provides superior pressure redistribution and significantly reduced peak pressure compared with standard foam and silicone dressings	

**Table 6:** Examples of commercially available hydrogels.

Commercial Product	Dressing Type	Observations	Refs.
Activheal Hydrogel	Amorphous hydrogel	Helps soften and hydrate eschar	[36,62]
		Provides a moist environment that facilitates healing	
		Effective in the debridement of necrotic, dry, or sloughy wounds	
		Suitable for light to medium exuding wounds	
		Can remain in situ for up to three days	
		Can be applied to varying depths of wounds	
		Requires a secondary dressing to hold it in place	
AquaClear	Sheet hydrogel	Active moisture-release system	[29,62,71]
		Maintains an optimal moisture balance that aids in healing	
		Promotes re-epithelialization	
		Occludes the wound without the need for a secondary dressing	
		Suitable for various ulcers, burns, and traumatic wounds	
Flaminal	Antimicrobial hydrogel	Embedded with an enzyme system that forms free radicals that kill bacteria by destroying their cell wall	[32,72]
		Wound exudate is absorbed in the hydrated form of the dressing	
		Ensures continuous debridement of dry scab and necrotic tissues	
		Suitable for burn wounds	
Hydrosorb	Sheet hydrogel	Suitable for keeping granulation tissue and young epithelium moist	[32,62,73]
		Provides a cushioning effect for wound protection	
		Soothing and cooling effect on superficial burns	
		Occludes the wound without the need for a secondary dressing	
HydroTac	Sheet hydrogel	Actively releases moisture and increases growth factor concentration	[32,62,74]
		Stimulates epithelial wound closure	
		Has an air-permeable, waterproof, and bacteria-proof film backing	
		Effective in removing devitalized tissue	
		Occludes the wound without the need for a secondary dressing	
IntraSite	Thick sterile hydrogel	Moderate elastic properties	[32,62,75]
		Improved patient comfort	
		Adequately rehydrates devitalized tissue	
		Promotes autolytic debridement in necrotic or sloughy wounds	
		Can be used on infected wounds due to its bacteriostatic and fungistatic activity	
		Easy application on the wounded area	
		Suitable for leg ulcers, pressure ulcers, and surgical wounds	
Iodozyme	Two-layer hydrogel	Exerts antimicrobial activity through iodine release	[32,58,76]
		Suitable for patients with chronic infection or bacterial bioburden	
Oxyzyme	Two-layer hydrogel	Provides enzyme-activated in situ oxygen production	[32,58,76]
		Impedes microbial growth through iodine release	
		Suitable for treating chronic wounds	
Solosite	Amorphous thin preserved hydrogel	Can be applied to fill a deep wound with irregular contours	[32,76]
		Suitable for low to moderately exuding wounds	
		Can be used for treating pressure injuries, sinuses, and cavity wounds	

**Table 7:** Examples of commercially available alginate dressings.

Commercial Product	Dressing Type	Observations	Refs.
Algiderm	Guluronic acid-rich alginate dressing	Induces a strong gel formation	[88]
		Excellent dressing integrity	
		Can absorb 20 times its weight in exudate	
Curasorb	Guluronic acid-rich calcium alginate dressing	Needled non-woven structure	[88,89]
		Induces a strong gel formation	
		Excellent dressing integrity	
Kaltostat	Guluronic acid-rich calcium alginate dressing	Forms a firm hydrophilic gel over the wound, ensuring a moist warm environment	[35,36]
		May be applied to bleeding wounds due to its hemostatic properties	
		Suitable for moderate to high exuding wounds	
		Can be used also for infected wounds, but necessitates more frequent change	
		Requires a secondary dressing to hold it in place	
Seasorb	Mannuronate-rich alginate dressing	Forms a soft flexible gel upon hydration	[32,35,84,88]
		Limits wound secretions	
		Minimizes bacterial contamination	
		Suitable for burns, donor sites, diabetic, leg, and pressure ulcers	
Sorbsan	Mannuronate-rich calcium alginate dressing	Unneedled pressure-rolled structure	[32,35,88,89]
		Forms a soft fragile gel	
		Disintegrates rapidly compared with other alginates	
		Significant beneficial effects on leg ulcers	
		Can be used on burns, donor sites, pressure ulcers. and surgical wounds	
Tegagel	Mannuronate-rich calcium alginate dressing	Hydroentangled nonwoven structure	[89]
		As the fibers are closely compressed, fluid diffusion is more difficult than in other alginates	
		High degree of wet integrity	

## Data Availability

Not applicable.
